# Is Postpartum Bipolar Disorder a Distinct Diagnostic Entity?

**DOI:** 10.1111/bdi.70032

**Published:** 2025-05-01

**Authors:** Verinder Sharma, Katelyn N. Wood, Boseok Cha, Dwight Mazmanian

**Affiliations:** ^1^ Department of Psychiatry Western University London Ontario Canada; ^2^ Department of Obstetrics & Gynecology Western University London Ontario Canada; ^3^ Parkwood Institute Mental Health St. Joseph's Health Care London Ontario Canada; ^4^ Department of Anatomy and Cell Biology Western University London Ontario Canada; ^5^ Department of Psychiatry Gyeongsang National University College of Medicine Jinju Republic of Korea; ^6^ Department of Psychology Lakehead University Thunder Bay Ontario Canada

Since the mid‐19th century when Louis‐Victor Marcé's monograph describing the effect of pregnancy, postpartum, and lactation on mental illness was published, the question of whether serious mental illness in the postpartum period is a distinct diagnostic entity has been intensely debated. Marcé noted that each symptom of postpartum mental illness could be found in non‐postpartum cases; however, the syndromes of postpartum illnesses were different from those of nonpuerperal mental illnesses. While DSM does not consider postpartum illnesses as separate entities, the unofficial yet popular classification promulgates the use of terms such as postpartum depression and postpartum psychosis. Until recently, the discussion about the diagnosis of postpartum disorders focused mainly on the distinctiveness of postpartum depression. Evidence for postpartum depression being a separate entity is mixed and depends largely on the definition of the postpartum period. Depression occurring later in the postpartum period appears to be more like depression occurring outside the postpartum period. However, depression in the early postpartum period (usually the first few weeks after delivery) may be a distinct entity, likely due to its later association with bipolar disorder—that is, the subsequent emergence of bipolar symptoms. While childbirth is a well‐known trigger of bipolar disorder in females, there is a lack of information about how it affects the symptom profile, course, and treatment response. Revisiting the debate about whether bipolar disorder in the postpartum period is different from its non‐postpartum counterpart is crucially important to improve the detection, diagnosis, and treatment of postpartum bipolar disorder. Due to the common practice of diagnosing patients with mood episodes and psychotic features as postpartum psychosis, it is unclear how commonly bipolar disorder begins with a manic or mixed episode in the postpartum period. Psychiatric disorders occurring immediately after delivery can be harbingers of bipolar disorder in some cases. Psychiatric disorders that morph into or are followed by bipolar disorder are more likely to have a first onset postpartum or an onset immediately after delivery than disorders occurring later in the postpartum period [1].

Studies on the phenomenology of postpartum manic episodes have provided mixed results. Compared with their non‐postpartum counterparts, postpartum manic episodes are more likely to be characterized by depression, anxiety, perplexity, mood lability, lassitude, and disorientation [2]. A within‐subject study retrospectively compared symptom profiles of postpartum and non‐postpartum manic episodes [3]. Manic episodes in the postpartum period had significantly fewer manic symptoms but more depressive symptoms. The duration of hospitalizations and short‐term outcomes were similar. Psychotic symptoms are common in females with postpartum manic, mixed, or depressive episodes; however, studies comparing their prevalence in postpartum and non‐postpartum populations are lacking. Depression is the most common occurrence postpartum, but mothers are also at an exceptionally high risk of recurrence of manic and mixed episodes [4]. First‐onset depression in the postpartum period is more likely to be characterized by higher rates of manic symptoms in first‐degree relatives, psychotic symptoms, atypical features, mixed depression, younger age at onset, and antidepressant‐induced hypo/mania compared with non‐postpartum onset depression [5]. There are no within‐subject or between‐subject studies on bipolar postpartum depression.

As mentioned, childbirth is considered a potent trigger of bipolar disorder; however, approximately 65% of females with the disorder do not have a postpartum recurrence. For those with a history of a bipolar mood episode postpartum, the episode does not recur after each delivery. Also, postpartum recurrences are not always true to type and can be replaced by the onset or recurrence of non‐affective illnesses, such as anxiety disorders or substance use disorders. Anxiety and substance use disorders commonly accompany bipolar disorder; however, their impact on the profile of bipolar mood episodes in the postpartum period has not been studied.

Some but not all studies have reported a better prognosis for bipolar disorder with postpartum onset compared with bipolar disorder with non‐postpartum onset. In general, studies comparing the course of bipolar disorder with or without a history of postpartum mood episodes have not found significant differences in clinical features, functioning, or severity. Sometimes the mood episodes are limited to the postpartum period, but non‐postpartum recurrences appear to be a rule rather than an exception. There is some consensus that females who do not have a mood episode after childbirth but develop bipolar disorder later in life have a worse prognosis.

Several factors such as increased stress, sleep loss, and hormonal changes may underlie the pathoplastic effect of childbirth; however, childbirth is not unique in this aspect because other reproductive events, especially menarche and menstrual periods, also affect the illness course of bipolar disorder, albeit to a lesser extent. Developmental phases also affect the clinical picture. For example, irritability, aggression, and hyperactivity are common during adolescence. Familial (probably genetic) factors have been implicated in susceptibility to puerperal episodes in females with bipolar I disorder, but studies of bipolar I and II populations have not found significant differences in the family histories of females with or without postpartum onset.

Due to the lack of controlled data on the prevention or acute treatment of bipolar mood episodes postpartum, it is unclear whether the medications generally considered effective for bipolar disorder are also effective in the postpartum period. There is preliminary evidence that lithium is effective in preventing mood episodes, especially in those with lithium‐responsive bipolar I disorder. Divalproex is not significantly more effective than monitoring without the drug for the prevention of postpartum episodes of bipolar disorder. Atypical antipsychotics, especially olanzapine, may be effective in preventing recurrences in females with bipolar I disorder. The treatment of depressive episodes with postpartum onset immediately after delivery poses unique challenges, especially in cases where there is no known family history of bipolar disorder. Antidepressants are generally prescribed for major depressive episodes, including in the postpartum period, but their use in some postpartum women may trigger hypo/manic or mixed symptoms. Atypical antipsychotics alone or in combination with medications such as lamotrigine or lithium may be preferable options for mothers who experience the first onset of depression immediately after delivery.

To summarize, there is some evidence that childbirth has a pathoplastic effect on the course of bipolar disorder, including its onset, polarity, and symptom profile. Since most women with bipolar postpartum episodes have a preexisting bipolar disorder, it can be argued that postpartum bipolar disorder does not constitute a distinct subgroup—at least to the extent of our current knowledge. While we await the results of future studies to clarify the short‐ and long‐term effects of childbirth on the course of the disorder, it is reasonable to conclude that bipolar disorder in the postpartum period is neither the same as its non‐postpartum counterpart nor distinct, but similar. The DSM peripartum onset specifier does not distinguish between symptoms of postpartum versus non‐postpartum bipolar disorder but allows the use of specifiers such as mixed features or anxious distress to elucidate the symptom profile of hypomanic, manic, and depressive episodes. Since mixed features associated with a major depressive episode are a risk factor for the development of bipolar I or II disorder, a detailed review of the timing of onset, current symptom profile, and safety issues is necessary for planning and monitoring the response to treatment. Controlled studies are needed on the pharmacological treatment of postpartum bipolar mood episodes in the future.

## Data Availability

The authors have nothing to report.
